# Mining the Unknown: A Systems Approach to Metabolite Identification Combining Genetic and Metabolic Information

**DOI:** 10.1371/journal.pgen.1003005

**Published:** 2012-10-18

**Authors:** Jan Krumsiek, Karsten Suhre, Anne M. Evans, Matthew W. Mitchell, Robert P. Mohney, Michael V. Milburn, Brigitte Wägele, Werner Römisch-Margl, Thomas Illig, Jerzy Adamski, Christian Gieger, Fabian J. Theis, Gabi Kastenmüller

**Affiliations:** 1 Institute of Bioinformatics and Systems Biology, Helmholtz Zentrum München, Neuherberg, Germany; 2 Department of Physiology and Biophysics, Weill Cornell Medical College in Qatar, Education City, Qatar Foundation, Doha, Qatar; 3 Metabolon, Research Triangle Park, North Carolina, United States of America; 4 Department of Genome-Oriented Bioinformatics, Life and Food Science Center Weihenstephan, Technische Universität München, Freising, Germany; 5 Research Unit of Molecular Epidemiology, Helmholtz Zentrum München, Neuherberg, Germany; 6 Biobank of the Hanover Medical School, Hanover Medical School, Hanover, Germany; 7 Institute of Experimental Genetics, Genome Analysis Center, Helmholtz Zentrum München, Neuherberg, Germany; 8 Lehrstuhl für Experimentelle Genetik, Technische Universität München, Freising-Weihenstephan, Germany; 9 Institute of Epidemiology, Helmholtz Zentrum München, Neuherberg, Germany; 10 Department of Mathematics, Technische Universität München, Garching, Germany; University of Oxford, United Kingdom

## Abstract

Recent genome-wide association studies (GWAS) with metabolomics data linked genetic variation in the human genome to differences in individual metabolite levels. A strong relevance of this metabolic individuality for biomedical and pharmaceutical research has been reported. However, a considerable amount of the molecules currently quantified by modern metabolomics techniques are chemically unidentified. The identification of these *“*unknown metabolites*”* is still a demanding and intricate task, limiting their usability as functional markers of metabolic processes. As a consequence, previous GWAS largely ignored unknown metabolites as metabolic traits for the analysis. Here we present a systems-level approach that combines genome-wide association analysis and Gaussian graphical modeling with metabolomics to predict the identity of the unknown metabolites. We apply our method to original data of 517 metabolic traits, of which 225 are unknowns, and genotyping information on 655,658 genetic variants, measured in 1,768 human blood samples. We report previously undescribed genotype–metabotype associations for six distinct gene loci (SLC22A2, COMT, CYP3A5, CYP2C18, GBA3, UGT3A1) and one locus not related to any known gene (rs12413935). Overlaying the inferred genetic associations, metabolic networks, and knowledge-based pathway information, we derive testable hypotheses on the biochemical identities of 106 unknown metabolites. As a proof of principle, we experimentally confirm nine concrete predictions. We demonstrate the benefit of our method for the functional interpretation of previous metabolomics biomarker studies on liver detoxification, hypertension, and insulin resistance. Our approach is generic in nature and can be directly transferred to metabolomics data from different experimental platforms.

## Author Summary

Genome-wide association studies on metabolomics data have demonstrated that genetic variation in metabolic enzymes and transporters leads to concentration changes in the respective metabolite levels. The conventional goal of these studies is the detection of novel interactions between the genome and the metabolic system, providing valuable insights for both basic research as well as clinical applications. In this study, we borrow the metabolomics GWAS concept for a novel, entirely different purpose. Metabolite measurements frequently produce signals where a certain substance can be reliably detected in the sample, but it has not yet been elucidated which specific metabolite this signal actually represents. The concept is comparable to a fingerprint: each one is uniquely identifiable, but as long as it is not registered in a database one cannot tell to whom this fingerprint belongs. Obviously, this issue tremendously reduces the usability of a metabolomics analyses. The genetic associations of such an “unknown,” however, give us concrete evidence of the metabolic pathway this substance is most probably involved in. Moreover, we complement the approach with a specific measure of correlation between metabolites, providing further evidence of the metabolic processes of the unknown. For a number of cases, this even allows for a concrete identity prediction, which we then experimentally validate in the lab.

## Introduction

Recently, genome-wide association studies (GWAS) on metabolic quantitative traits have proven valuable tools to uncover the genetically determined metabolic individuality in the general population [1]–[5]. Interestingly, a great portion of the genetic loci that were found to significantly associate with levels of specific metabolites are within or in close proximity to metabolic enzymes or transporters with known disease or pharmaceutical relevance. Moreover, compared to GWAS with clinical endpoints the effect sizes of the genotypes are exceptionally high.

The number and type of the metabolic features that went into these GWAS was mainly defined by the metabolomics techniques used: Gieger et al. [1] and Illig et al. [2] used a targeted mass spectrometry (MS)-based approach giving access to the concentrations of 363 and 163 metabolites, respectively. Suhre et al. [3] and Nicholson et al. [4] applied untargeted nuclear magnetic resonance (NMR) based metabolomics techniques, yielding 59 metabolites that had been identified in the spectra prior to the GWAS and 579 manually selected peaks from the spectra, respectively. In Suhre et al. [5], 276 metabolites from an untargeted MS-based approach were analyzed.

While these previous GWAS focused on metabolic features with known identity, untargeted metabolomics approaches additionally provide quantifications of so-called “*unknown metabolites”*. An unknown metabolite is a small molecule that can reproducibly be detected and quantified in a metabolomics experiment, but whose chemical identity has not been elucidated yet. In an experiment using liquid chromatography (LC) coupled to MS, such an unknown would be defined by a specific retention time, one or multiple masses (e.g. from adducts), and a characteristic fragmentation pattern of the primary ion(s). An unknown observed by NMR spectroscopy would correspond to a pattern in the chemical shifts. Unknowns may constitute previously undocumented small molecules, such as rare xenobiotics or secondary products of metabolism, or they may represent molecules from established pathways which could not be assigned using current libraries of MS fragmentation patterns [6], [7] or NMR reference spectra [8].

The impact of unknown metabolites for biomedical research has been shown in recent metabolomics-based discovery studies of novel biomarkers for diseases and various disease-causing conditions. This includes studies investigating altered metabolite levels in blood for insulin resistance [9], type 2 diabetes [10], and heart disorders [11]. A considerable number of high-ranking hits reported in these biomarker studies represent unknown metabolites. As long as their chemical identities are not clarified the usability of unknown metabolites as functional biomarkers for further investigations and clinical applications is rather limited.

In mass-spectrometry-based metabolomics approaches, the assignment of chemical identity usually involves the interpretation and comparison of experiment-specific parameters, such as accurate masses, isotope distributions, fragmentation patterns, and chromatography retention times [12]–[14]. Various computer-based methods have been developed to automate this process. For example, Rasche and colleagues [15] elucidated structural information of unknown metabolites in a mass-spectrometry setup using a graph-theoretical approach. Their approach attempts to reconstruct the underlying fragmentation tree based on mass-spectra at varying collision energies. Other authors excluded false candidates for a given unknown by comparing observed and predicted chromatography retention times [16], [17], or by the automatic determination of sum formulas from isotope distributions [18]. Furthermore, Gipson et al. [19] and Weber et al. [20] integrated public metabolic pathway information with correlating peak pairs in order to facilitate metabolite identification. However, these methods might not be applicable for high-throughput metabolomics datasets that have been produced in a fee-for-service manner, since the mass spectra as such might not be readily available.

**Figure 1:**
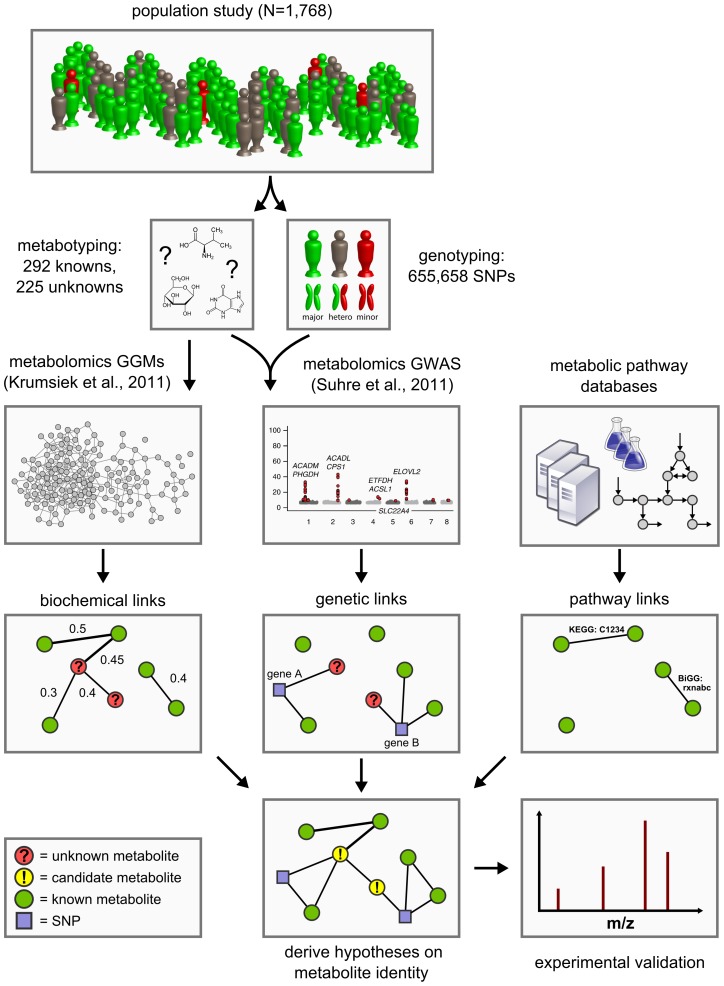
Data integration workflow for the systematic classification of unknown metabolites. We combine high-throughput metabolomics and genotyping data in Gaussian graphical models (GGMs) [21] and in genome-wide association studies (GWAS) [5] in order to produce testable predictions of the unknown metabolites' identities. These hypotheses are then subject to experimental verification by mass-spectrometry. Six such cases have been fully worked through and are presented in Table 3.

Approaching the problem from a conceptually different perspective, we here present a novel functional metabolomics method to predict the identities of unknown metabolites using a systems biological framework. By combining high-throughput genotyping data, metabolomics data, and literature-derived metabolic pathway information, we generate testable hypotheses on the metabolite identities based solely on the obtained metabolite quantifications (Figure 1). No further experiment-specific data such as retention times, isotope patterns and fragmentation patterns are required for this analysis.

The concept of our approach is based on the following observations from our previous work on genome-wide association studies and Gaussian graphical modeling (GGM) with metabolomics: We showed that GWAS with metabolic traits can reveal functional relationships between genetic loci encoding metabolic enzymes and metabolite concentration levels in the blood [1]–[3], [5]. A genetic variant can alter, for instance, the expression levels of mRNAs or affect the properties of the respective enzymes through changes of the protein sequence (e.g. enzyme activity, substrate specificity). Moreover, we found that GGMs, which are based on partial correlation coefficients, can identify biochemically related metabolites from high-throughput metabolomics data alone [21], [22]. These observations suggest that if an unknown compound displays a similar statistical association with a genetic locus in a GWAS or a known metabolite in a GGM, then this may provide specific information of where it is located in the metabolic network. Based on this information we can then derive testable hypotheses on the biochemical identity of the unknown metabolite. This annotation idea parallels classical concepts from functional genomics, where, for instance, co-expression between RNA transcripts is used to predict the function of poorly characterized genes [23], [24].

The manuscript is organized as follows: We first conduct a full genome-wide association study on 655,658 genotyped SNPs with concentrations of 225 unknown metabolites using fasting blood serum samples from a large German population cohort (n = 1768) [25]. We thereby extend our previous work on known metabolites [5] to a GWAS with hitherto unpublished unknown metabolic traits. We then compute a Gaussian graphical model including both known and unknown metabolites. In a third step, we integrate the results of the GWAS and GGM computations and combine them with metabolic pathway information from public databases to derive predictions for a total of 106 unknown metabolites. In order to validate the approach, we investigate six distinct cases, in which we derive specific identity predictions for a total of nine unknown metabolites, which we then confirm experimentally. Finally, we discuss the relevance of newly discovered genetic loci and unknown identity predictions in the context of existing disease biomarker discovery and pharmacogenomics studies.

All GWAS and GGM results, unknown metabolite classifications and pathway annotations are available as spreadsheets and in .graphml format in Dataset S1 or from our study website at http://cmb.helmholtz-muenchen.de/unknowns.

## Results

### Genetic association links unknown metabolites to functionally related genes

In the first step of our analysis, we conducted a GWAS with the concentrations of known and unknown metabolites, testing a total of 655,658 genotyped SNPs from the KORA cohort for association. Thus, in addition to the unknown metabolite data, we included the association data for known metabolites from our previous study [5] into the present analysis. Unknown metabolites are uniquely labeled in the format “X-12345”, which are identical throughout all published studies that use the Metabolon platform.

**Figure 2:**
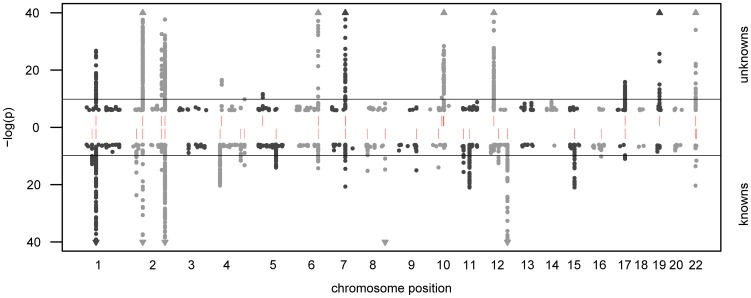
Manhattan plot of genetic association. The strength of association for known (bottom) and unknown (top) metabolites is indicated as the negative logarithm of the p-value for the linear model (see Methods). Only metabolite-SNP associations with p-values below 10^−6^ are plotted (grey circles). Triangles represent metabolite-SNP associations with p-values below 10^−40^. Horizontal lines indicate the threshold for genome-wide significance (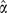 = 1.6×10^−10^ corresponding to α = 0.05 after Bonferroni correction); red vertical dashes indicate loci at which this threshold is attained.

In total, we observe 34 distinct loci that display metabolite associations at a genome-wide significance level (Figure 2 and Dataset S1). Out of these 34 loci, 15 associate with at least one unknown compound. For 12 loci, an unknown compound constitutes the strongest association of all tested compounds. From the 213 unknown metabolites analyzed (see Methods for the determination of this metabolite subset), 28 show at least one genome-wide significant hit. These 28 associations at the 15 loci are presented in Table 1 along with all previously described GWAS hits to metabolic traits or other endpoints. Associating traits were determined from the GWAS catalog [26] for SNPs in LD (r^2^≥0.5) with the respective lead SNP. Seven of the 15 loci (SLC22A2, COMT, CYP3A5, CYP2C18, GBA3, UGT3A1, rs12413935) have not been described in GWAS with metabolic traits before and thus represent new genetic loci of metabolic individuality. Interestingly, genetic variants in strong LD with CYP2C18 have been reported to associate with warfarin maintenance dose [27].

**Table 1:** Genome-wide significant associations (p<1.6×10^−10^) involving unknown metabolites.

Locus	Locus Info	Lead-SNP	Chr	BP	Metabolite	P-value	Published GWAS hits(metabolic traits)	Published GWAS hits(further phenotypes)
PYROXD2	pyridine nucleotide-disulfide oxidoreductase domain 2	rs4488133	10	100149126	X-12092	2.2×10^−281^	trimethylamine (urine)/dimethylamine (plasma) [4]	-
					X-12093	1.4×10^−27^		
SLCO1B1	organic anion transporter family, bile acids	rs4149056	12	21269288	X-11529	3.3×10^−81^	eicosenoate/tetradecanedioate [5]bilirubin [49], [50]	statin response [51]
					X-11538	1.4×10^−37^		
					X-13429	4.9×10^−22^		
					X-12063	5.2×10^−20^		
					X-12456	8.4×10^−17^		
					X-14626	2.1×10^−13^		
SLC22A2	solute carrier family 22 (organic cation transporter), member 2	rs316020	6	160589071	X-12798	1.7×10^−72^	*New*	-
NAT8	N-acetyltransferase 8	rs7598396	2	73672444	X-12510	1.5×10^−56^	N-acetylornithine [5], creatinine [52]	chronic kidney disease [53]
					X-11787	3.0×10^−37^		
					X-12093	8.9×10^−22^		
COMT	catechol-O-methyltransferase	rs4680	22	18331271	X-11593	1.1×10^−48^	*New*	-
					X-01911	5.8×10^−11^		
CYP3A5	cytochrome P450, family 3, subfamily A, polypeptide 5	rs10242455	7	99078115	X-12063	1.5×10^−45^	*New*	-
SULT2A1	sulfotransferase family, cytosolic, 2A, dehydroepiandrosterone-preferring	rs296391	19	53060346	X-11440	1.7×10^−43^	dehydroepiandrosterone sulfate [54]	-
					X-11244	2.1×10^−26^		
UGT1A	UDP glucuronosyltransferase 1 family, polypeptide A complex locus	rs6742078	2	234333309	X-11530	2.1×10^−38^	bilirubin (E,E)/oleoylcarnitine [5]bilirubin [49], [50], [55], [56]	circulating cell-free DNA [57]
					X-11441	5.6×10^−30^		
					X-11793	2.6×10^−26^		
					X-11442	1.2×10^−25^		
ACADL	acyl-CoA dehydrogenase, long-chain	rs2286963	2	210768295	X-13431	2.7×10^−33^	C9/C10:2 [2]	
ACADM	acyl-CoA dehydrogenase, medium-chain	rs12134854	1	75879263	X-11421	1.9×10^−27^	C12/C8 [1], [2], [5], C12/C10 [2]hexanoylcarnitine/oleate [3], [5]	-
CYP2C18	cytochrome P450, family 2, subfamily C, polypeptide 18	rs7896133	10	96454720	X-11787	4.0×10^−26^	*New*	warfarin maintenance dose [27]
GBA3	glucosidase, beta, acid 3 (cytosolic)	rs358231	4	22429602	X-11799	2.9×10^−17^	*New*	-
ACE	angiotensin I converting enzyme (peptidyl-dipeptidase A) 1	rs4343	17	58917190	X-14189	1.5×10^−16^	aspartylphenylalanine [5]	angiotensin-converting enzyme activity [28]
					X-14208	4.6×10^−15^		
					X-14205	4.0×10^−14^		
					X-14304	2.7×10^−12^		
UGT3A1	UDP glycosyltransferase 3 family, polypeptide A1	rs13358334	5	36025563	X-11445	2.4×10^−12^	*New*	-
—	[no known gene locus]	rs12413935	10	85443900	X-06226	4.0×10^−11^	*New*	-

We observe associations at 15 genetic loci that involve genes from various biological processes. Note that most of these genes code for proteins that are related to metabolic activities in the body, thereby providing information that allows to derive concrete hypotheses on the biochemical identity of each unknown. Previously published associations with known metabolites and other phenotypic traits (derived from the GWAS catalog [26]) provide further evidence on specific parts of a pathway in which the unknown might be involved. Chromosomal locations are reported with respect to the positive strand of human genome build 36.1.

In our previous GWAS with metabolic traits, we observed that metabolites associating with genetic variants in or near enzymes are likely to be functionally linked to these proteins. A SNP with detectable effects on the metabolome will, for instance, alter expression levels of mRNAs, or affect the properties of the respective enzymes (e.g. enzyme activity, substrate specificity) through modifications of the protein sequence. As an example of the latter case, the SNP rs4343 in the angiotensin converting enzyme (ACE) encoding gene was found to be associated with the activity of the enzyme [28] (See Table 1). To estimate the contribution of the first case, we compared our significant SNPs with expression quantitative trait loci (eQTLs) from published GWAS with expression levels. To this end, we queried the Genotype-Tissue Expression (GTEx) browser, an online eQTL database of the NIH GTEx roadmap project, which stores eQTL results for multiple human tissues (liver, lymphoblastoid, brain) [29]. For seven SNPs in three distinct loci (PYROXD2, CYP3A5, SPPL3), we found significant *cis*-eQTLs (p-value<2.7×10^−9^, see Methods) in GTEx. All identified eQTLs with p-values below 10^−5^ are listed in Dataset S1.

Based on the observation that SNPs in or in the vicinity of enzymes are mostly associating with functionally related metabolites in case of the knowns, we used the GWAS data to derive hypotheses on the potential identity of the respective unknowns. For instance, the SNP rs296391 in close proximity to the SULT2A1 gene (*sulfotransferase family, cytosolic, 2A, dehydroepiandrosterone DHEA-preferring*) strongly associates with the concentrations of the unknown metabolites X-11440 and X-11244 (p = 1.7×10^−43^ and p = 2.1×10^−26^, respectively). The enzyme encoded by SULT2A1, a *bile salt sulfotransferase*, converts steroids and bile acids into water-soluble sulfate conjugates for excretion [30]. Thus, we may speculate that X-11440 and X-11244 are biochemically related to steroids, bile acids, or water-soluble sulfate conjugates. Additional insights can be gained from genetic associations that involve both known and unknown metabolites. For instance, X-12510, X-11787, X-12093 and N-acetylornithine strongly associate with genetic variation at the NAT8 locus. NAT8 encodes the protein *N-acetyltransferase 8*. In this case, we may speculate that the unknowns represent similar substrates or products of the N-acetylation processes linked to this enzyme. Finally, we can link the results obtained here with results from other GWAS on metabolic traits. For example, the unknown metabolite X-13431 associates with a genetic variant in the ACADL (*acyl-CoA dehydrogenase, long-chain)* gene. This locus does not associate with any other metabolite in the present study, but was previously reported to associate with the medium-chain length carnitines C9 and C10:1 [1], [2]. Proteins from the ACAD family catalyze rate-limiting reactions in the β-oxidation pathway which generally associate with carnitines. This observation suggests that X-13431 may be a member of this medium-chain length carnitine family. These examples demonstrate that concrete information on the biochemical identity of unknown metabolites can be derived from our experimental dataset by using the GWAS approach.

### Gaussian graphical modeling provides a biochemical context for unknown metabolites

**Figure 3:**
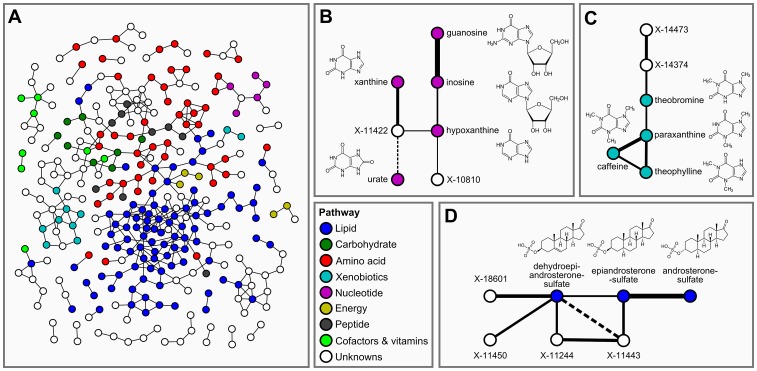
Gaussian graphical modeling. GGMs embed unknown metabolites into their biochemical context. A: Complete network presentation of partial correlations that are significantly different from zero at α = 0.05 after Bonferroni correction. The unknown metabolites are spread over the entire network and are involved in various metabolic pathways. B–D: Selected high-scoring sub-networks. We observe that GGM edges directly correspond to chemical reactions which alter specific chemical groups (e.g. carbonyl groups and methyl groups). Solid lines denote positive partial correlation. Dashed lines indicate negative partial correlations. Line widths represent partial correlation strengths.

In the second step of our analysis we focused solely on intrinsic relations between the measured metabolites and, in particular, on associations between known and unknown compounds. To this end, we applied Gaussian graphical models (GGMs), which we have previously shown to be able to reconstruct pathways involving directly related metabolites from cross-sectional blood serum metabolomics data [21], [22]. GGMs are based on partial correlation coefficients, that is, correlations between pairs of metabolites corrected for the effects of all remaining metabolites. Each known metabolite is annotated with a “super-pathway” corresponding to its general metabolic class, and a “sub-pathway” representing more specific metabolic pathways (see Dataset S1). In order to obtain a dataset that is independent of our genetic analysis, and to avoid circular arguments, co-variations in metabolite concentrations that are due to association with genetic variants (SNPs) were specifically removed from the data (see GGM methods for further details). A partial correlation was included in the model if it was significantly different from zero with α = 0.05 after Bonferroni correction, yielding a corrected significance level of 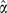 = 7.9×10^−7^ and an absolute partial correlation cutoff of ζ = 0.178. The resulting GGM consists of a total of 399 out of 62,835 theoretically possible edges (0.64% connectivity, Figure 3A). In line with our previous observations [21], metabolites tend to be strongly connected within their respective metabolic class, while links between different classes are rare (see Text S1). Inspecting the GGM in detail, we observe that the unknowns are tightly integrated within the network and connected to known compounds of various metabolic classes. This is reflected both in the overall network (Figure 3A, Text S1) and in the top list of high-scoring GGM edges (Table 2), where 18 of the 30 strongest partial correlations comprise at least one unknown metabolite. The highest partial correlation in the dataset actually involves a known-unknown metabolite pair, namely 3-indoxylsulfate and the unknown metabolite X-12405 (ζ = 0.840). For pairs of known metabolites, we consistently observe associations of biochemically related metabolites from various metabolic pathways, such as the metabolites inosine and guanosine (ζ = 0.798), which are involved in nucleotide metabolism, or androsterone sulfate and epiandrosterone sulfate (ζ = 0.755), which represent related steroid hormone metabolites. Other pathways with related metabolite pairs include amino acid metabolism, lipid metabolism, bile acid metabolism, and xanthine metabolism. Following our line of reasoning, correlating pairs of a known and an unknown metabolite then directly point to specific pathways of cellular metabolism on which the unknown metabolite may lie. The investigation of the sub-network structure around the unknown compounds provides additional biochemical context for that compound.

**Table 2:** Interpretation of top-ranking partial correlation coefficients (PCC>0.5).

Metabolite 1	Metabolite 2	ζ	Interpretation
X-11847	X-11849	0.901	biochemical link between two unknowns
3-indoxyl sulfate	X-12405	0.840	*tryptophan metabolism*
X-11452	X-12231	0.832	biochemical link between two unknowns
X-12094	X-12095	0.822	biochemical link between two unknowns
guanosine	inosine	0.798	nucleosides
X-11441	X-11442	0.760	biochemical link between two unknowns
androsterone sulfate	epiandrosterone sulfate	0.755	steroid sulfates
X-11537	X-11540	0.753	biochemical link between two unknowns
X-02269	X-11469	0.734	biochemical link between two unknowns
X-11204	X-11327	0.706	biochemical link between two unknowns
decanoylcarnitine	octanoylcarnitine	0.689	β-oxidation footprints
linoleamide (18:2n6)	oleamide	0.654	C18:1/C18:2 acylamides
3-methyl-2-oxovalerate	4-methyl-2-oxopentanoate	0.646	branched-chain amino acid degradation
catecholsulfate	X-12217	0.601	*catechol metabolism*
X-14189	X-14304	0.593	biochemical link between two unknowns
1,5-anhydroglucitol (1,5-AG)	X-12696	0.580	*sugar metabolism*
dehydroisoandrosterone sulfate (DHEA-S)	X-18601	0.575	*steroid hormones*
1-arachidonoylglycerophosphoethanolamine	X-12644	0.570	*phospholipids (PE)*
X-14208	X-14478	0.558	biochemical link between two unknowns
caffeine	paraxanthine	0.554	caffeine metabolism
X-11423	X-12749	0.549	biochemical link between two unknowns
1-linoleoylglycerophosphocholine	2-palmitoylglycerophosphocholine	0.544	phospholipids (PC)
piperine	X-01911	0.526	*amino acid-derived alkaloids*
2-hydroxypalmitate	2-hydroxystearate	0.523	hydroxy fatty acids
X-14056	X-14057	0.519	biochemical link between two unknowns
3-methyl-2-oxovalerate	isoleucine	0.514	isoleucine degradation
X-11244	X-11443	0.510	biochemical link between two unknowns
urea	X-09706	0.506	*urea metabolism*
isoleucine	leucine	0.506	branched-chain amino acids
1-arachidonoylglycerophosphoethanolamine	1-linoleoylglycerophosphoethanolamine	0.502	phospholipids (PE)

Connections between two known metabolites indicate a direct metabolic relationship, e.g. between purines (guanosine/inosine) or steroid hormones (androsterone sulfate/epiandrosterone sulfate). A link between a known and an unknown compound therefore provides evidence for a shared metabolic pathway. For instance, the link between 3-indoxylsulfate and X-12405 suggests a role of this unknown in tryptophan metabolism. Abbreviations: PC = phosphatidylcholine, PE = phosphatidylethanolamine, ζ = partial correlation coefficient. *Italic* text represents hypothetical known-unknown connections.

We selected four high-scoring sub-networks in the GGM to show that this concept is indeed applicable to real data. The first two of these sub-networks consist of a series of intermediate compounds from purine metabolism, including guanosine, inosine, xanthine derivatives and urate (Figure 3B and 3C). In these cases, one can actually follow the addition and removal of chemical groups by following the edges in the GGM network: Most edges in these sub-networks correspond to the change of either a single methyl group at the purine double-ring structure or to the removal of a ribose residue in the reaction from nucleosides to xanthine variants. While the compounds in both sub-networks appear structurally similar, the distinction into two groups by the GGM is indeed biochemically sound. The metabolites in Figure 3B correspond to endogenous substances in the nucleoside pathway, whereas the molecules in Figure 3C relate to signals from xenobiotic metabolism of drugs and caffeine. Here, the unknown metabolites X-11422 and X-10810, as well as X-14473 and X-14374 are prominently placed in the networks, making them direct targets for closer inspection with respect to endogenous xanthines and xenobiotics, respectively.

The third sub-network comprises three androsterone sulfate variants, which belong to the class of steroid hormones (Figure 3D). We observe direct GGM links between the unknowns X-11450, X-11244 and X-11443 with both dehydroepiandrosterone sulfate (DHEAS) and epiandrosterone sulfate, suggesting androsterone derivatives as likely candidates for these three metabolites. The fourth sub-network involves different stereoisomers of bilirubin, which is the degradation product of the oxygen transporter hemoglobin [31] (Figure S1). In this sub-network, we observe high partial correlations between the bilirubin variants and a series of unknown metabolites (X-11441, X-11530, X-11442, X-11793, X-11809, X-14056, and X-14057). The seven unknown compounds in this GGM sub-network are thus likely to be involved in hemoglobin degradation processes. Taken together, the examples confirm that further information on the biochemical identity of unknown metabolites can be extracted from GGM networks.

### Combining GGMs and GWAS allows deriving specific pathway annotations for unknown metabolites

The next step in our analysis was the integration of the GGM and GWAS approaches with general pathway information from external databases, in order to generate concrete predictions for the unknowns' metabolic pathway memberships. As a feasibility test, we first asked whether the local neighborhood of a metabolite in the GGM can be used to correctly predict its metabolic class. Using a majority-voting based classifier and subsequent permutation testing, we detected significant classification abilities (mean sensitivity 0.674, mean specificity 0.84, macro-averaged F_1_ score 0.72) far beyond random (p<10^−8^,). Detailed results can be found in Text S2. Note that we performed this approach only to demonstrate the systematic possibility to derive functional information from the GGM. The actual classification of the unknowns in the following will not be based on majority voting, but rather on the collection of all available functional information from GGM neighbors and GWAS hits.

**Figure 4:**
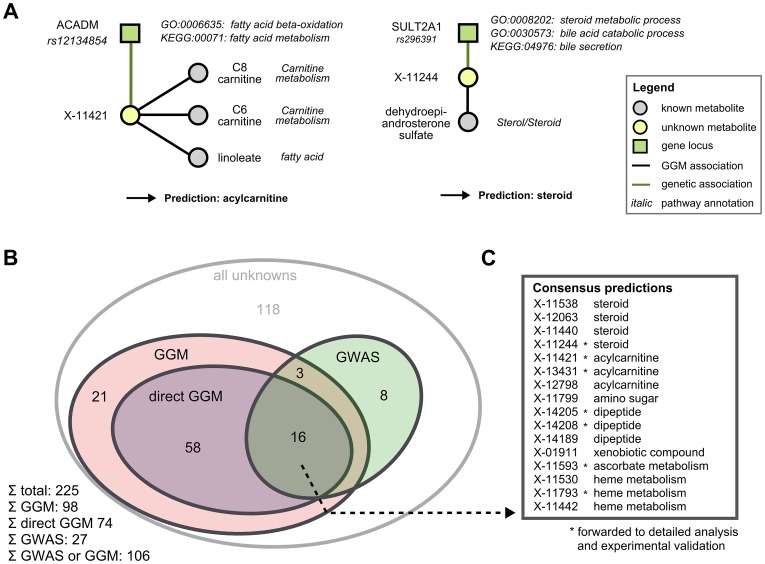
Semi-automatic prediction of unknown metabolite identities. A: Examples of how to determine pathway classifications based on the functional annotations of GGM and GWAS hits. We present two metabolites, X-11421 and X-11244, whose GGM and GWAS associations clearly point into carnitine and steroid metabolism, respectively. B: Overview of unknowns functionally annotated by both GGMs and the GWAS approach. ‘GGM’ refers to an unknown metabolite which is three or less steps away from a known metabolite in the GGM, whereas ‘direct GGM’ represents direct neighbors in the network. C: Pathway predictions for the 16 unknowns with both direct GGM and GWAs annotations. Unknowns marked with a star were subjected to in-depth analysis followed by experimental validation in the following.

We combined functional annotations for both GGM neighbors and GWAS hits for each unknown in order to derive specific pathway classifications. For unknowns that did not have a known metabolite neighbor in the GGM, we also investigated the 2- and 3-neighborhoods. Since these hits certainly represent weaker evidence than a direct GGM neighbor, we distinguish between ‘GGM hit’ and ‘direct GGM hit’ in the following. Functional annotations were obtained from three sources: (1) The *sub-pathway* assignment provided for each known metabolite in the GGM neighborhood, (2) the GO functional terms for the associated gene of all genome-wide significant GWAS hits, and (3) the KEGG pathways on which the associated genes lie. To the best of our knowledge, there is presently no consistent mapping between annotations from the different data sources available for both metabolites and genes, so we here had to perform the only non-automatic step in the analysis: By manual interpretation of different functional classes (Figure 4A), we derive a single consensus pathway annotation for a total of 106 of the unknown metabolites (Figure 4B). For 98 unknowns, we obtained annotations from the GGM network, with 74 of these hits representing direct GGM hits. From the 28 genetic hits introduced above, 27 were in a genetic region with gene annotation. Overlaying the direct edge GGM set and the GWAS set, we obtained 16 unknowns with both biochemical and genetic evidence (Figure 4C). A list of all functional evidence along with the respective predictions can be found in Table S1.

### Experimental validation of nine predictions in six distinct scenarios

In the following, we selected several unknowns that were forwarded to detailed analysis and experimental validation. Five cases were obtained from the set of 16 high-confidence predictions in the previous section, since the combined evidence from GWAS and GGMs provides rich functional annotations that allow to derive possible compound candidates. Moreover, in order to demonstrate the power of GGMs in the absence of genetic associations, we selected one further case (HETE) where publically available pathway information was systematically exploited. Experimental validations were performed by running pure candidate compounds on the LC-MS/MS platform. For cases where no pure compound was available, we determined exact molecule masses and revisited the retention times and fragmentation spectra.

**Figure 5:**
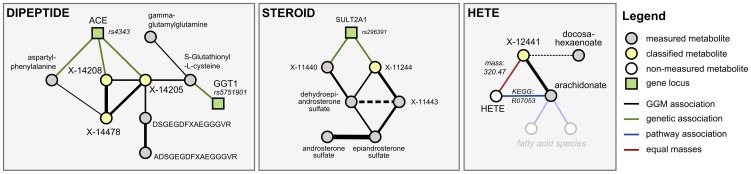
Detailed investigation of three scenarios (DIPEPTIDE, STEROID, and HETE). In order to generate concrete hypotheses on the unknowns' identities, we assembled all available information for each scenario. This includes biochemical edges from the GGM, genetic associations from the GWAS, pathway annotations as well as mass information. For details of the predicted identities, see Table 3 and main text. Similar figures for three further scenarios (CARNITINE, BILIRUBIN, and ASCORBATE) are available in Text S3.

We investigated six metabolic scenarios in-depth and attempted experimental confirmation of the respective predictions (Table 3). In the following, we discuss three example cases, termed DIPEPTIDE, STEROID, and HETE (Figure 5). Three further examples, named CARNITINE, BILIRUBIN, and ASCORBATE, are presented as Text S3. In the discussion of these scenarios we now use all available evidence, the metabolite correlations, genetic associations, biochemical data, and in addition the molecular masses reported with the known and unknown compounds (which do not represent exact masses at this point). Note that the presented scenarios represent the only cases where a detailed investigation has been attempted. Moreover, the candidate compounds mentioned in the following paragraphs and the supplementary material are the only compounds that have been experimentally tested (there are no negative results not reported in this text).

**Table 3:** Six specific scenarios and their experimental validations.

Scenario name	Unknowns	Evidence used	Prediction	Validated as
DIPEPTIDE	X-14208	GGM, genetics	Phe-Ser or Ser-Phe	Phe-Ser
	X-14205		Glu-Tyr or Try-Glu	α-Glu-Tyr
	X-14778		Phe-Phe	Phe-Phe
STEROID	X-11244	GGM, genetics	sulfated androsterone	androstene disulfate
HETE	X-12441	GGM, pathway	hydroxy-arachidonate (HETE)	12-HETE
CARNITINE	X-11421	GGM, genetics, pathway	carnitine species, with 6 to 10 carbon atoms	cis-4-decenoyl-carnitine
	X-13431			nonanoyl carnitine*
BILIRUBIN	X-11793	GGM, genetics	oxidized bilirubin variant	oxidized bilirubin variant*
ASCORBATE	X-11593	GGM, genetics, pathway	O-methylascorbate	O-methylascorbate*

We investigated six scenarios that included a total of nine unknown metabolites. The first three scenarios, DIPEPTIDE, STEROID, and HETE are discussed in the main text of this paper; the remaining three scenarios, CARNITINE, BILIRUBIN and ASCORBATE, are discussed in Text S3. Predictions marked by * are confirmed by exact mass, fragmentation pattern and chromatographic retention time; however, validation using a pure standard compound as a reference is pending since these compounds are presently commercially unavailable in pure form.

#### Scenario 1

Our first scenario, DIPEPTIDE, presents the prediction and successful validation of three unknown metabolites involved in short-peptide metabolism (Figure 5, left). In the GGM, we observe X-14205, X-14208 and X-14478 in close proximity to various dipeptides, to glutathione derivatives, and to two longer fibrinogen-related peptides. The primary pieces of genetic evidence for this case are the GGT1 locus, which shows a strong association to S-gluthathionyl-L-cysteine, and the ACE locus, which connects to aspartyl-phenylalanine, X-14205, and X-14208. GGT1 encodes for the protein γ-glutamyl transpeptidase, which transfers glutamyl-residues from glutathione in order to generate short-chain peptides [32]. This fits well into the network picture, since GGT1 is connected to the glutathione derivative, which in turn shares a GGM edge with γ-glutamyl-glutamine. ACE, on the other hand, encodes the angiotensin I converting enzyme, a peptidase that cleaves dipeptide fragments from angiotensin precursors and other functional oligopeptides. Since the biochemical and genetic evidence pointed us to short peptides, and dipeptides in particular, we enumerated all possible 400 ( = 20×20) combinatorial variants of dipeptides and checked the mass against the masses of the three unknowns under investigation. As an example, we shortened the list of candidates for X-14208 from 2,732 (ChemSpider search) to only 8 molecules, respectively.

**Figure 6:**
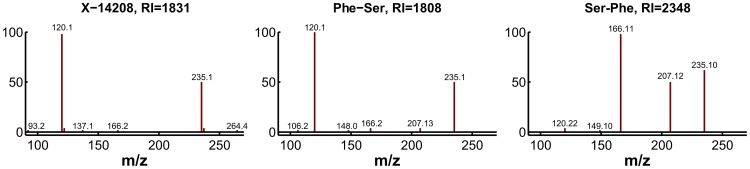
Experimental confirmation of X-14208 as phenylalanylserine. Two possible dipeptide variants were predicted and consequently tested. The fragmentation spectrum of the 253.1 m/z ion (positive mode) of the pure Phe-Ser matches that of the unknown compound, whereas the spectrum for pure Ser-Phe differs visibly. Moreover, the retention index (RI) of Phe-Ser is similar to the RI of X-14208, whereas that of Ser-Phe is significantly different.

For experimental validation, we first checked the plausibility of the candidates with respect to the fragmentation spectra and determined the exact masses. The accurate mass determined for X-14208 is 252.11172±0.001 Da, supporting the chemical formula C_12_H_16_N_2_O_4_. While the formula still matches more than 1,200 molecular structures, the prediction of this unknown as a dipeptide leaves only two candidate molecules, namely phenylalanylserine (Phe-Ser) and serylphenylalanine (Ser-Phe). Both variants were obtained from a commercial source and run on the LC-MS/MS platform. The retention index [33] and the fragmentation spectrum received for Phe-Ser matched the index and spectrum of X-14208, whereas Ser-Phe produced a clearly different spectrum (Figure 6). Thus, the identity of X-14208 was experimentally confirmed as the dipeptide phenylalanylserine. Importantly, using our integrated approach, we were able to identify X-14208 by only testing two candidate molecules. The other two unknowns, X-14205 and X-14478, were identified through similar experiments as α-glutamyltyrosine (α-Glu-Tyr) and phenylalanylphenylalanine (Phe-Phe), respectively.

#### Scenario 2

In the second scenario, STEROID, we investigated an unknown metabolite (X-11244) for which both GGM and GWAS data strongly indicate an identity related to steroid-hormone compounds: X-11244 is tightly linked via GGM edges to dehydroepiandrosterone sulfate and two other unknowns, which in turn connect to epiandrosterone sulfate and androsterone sulfate (Figure 5, middle). Furthermore, X-11244 displays a highly significant genetic association (p = 2.1×10^−26^) with rs296391, which lies in strong LD in the SULT2A1 gene locus. SULT2A1 encodes for a member of the *sulfotransferase family 2A, dehydroepiandrosterone-preferring*, further strengthening the metabolic context. Based on the GGM and GWAS results, we hypothesized that X-11244 is a steroid sulfate related to androstane.

Experimentally, the primary loss of a fragment with a nominal mass of 98 and the presence of an ion at 97 m/z observable in the fragmentation spectrum of X-11244 indicate the presence of at least one sulfate group in this unknown (Text S3). The exact mass determined for X-11244 supports the chemical formula C_19_H_30_O_8_S_2_. Querying ChemSpider for this chemical formula yields only four results, one of which corresponds to an androstene disulfate variant (ChemSpider ID 21403154). Analysis of several disulfated androstenes demonstrated similar retention times and fragmentation spectra. Among the tested variants, 4-androsten-3β,17β-disulfate showed the best match. Given that other isomers are also possible, which cannot necessarily be chromatographically resolved, we annotated X-11244 more generically as ‘androstene disulfate’.

#### Scenario 3

In the third scenario, HETE, we made explicit use of known biochemical interaction derived from three publically available pathway databases. We searched for cases, where an unknown shows GGM connections to known compounds for which a direct pathway interaction with a metabolite having the same mass as the unknown exists. Such cases are rare, but if present provide a strong argument for an unknown's identity. We selected the HETE scenario as an example for experimental validation in such a situation. The unknown metabolite X-12441 does not show any genome-wide significant SNP hits and only a single GGM neighbor: *cis-5,8,11,14-eicosatetraenoic acid* (arachidonate, Figure 5, right). Arachidonate constitutes pathway connections to several other lipid-related metabolites, including a variety of hydroxy-arachidonate variants (HETEs). These variants have the chemical formula C_20_H_32_O_3_ with a molecular weight of 320.2351 Da, matching the mass of the unknown. We thus hypothesized that X-12441 represents a specific HETE species.

Experimentally, the determination of the exact mass of the unknown further supported our hypothesis, as the accurate mass determined for X-12441 matches the chemical composition of HETE to a precision of ±0.002 Da. A number of HETE isoforms were experimentally tested, including the 5, 8, 9, 11, 12 and 15 isoforms. All isoforms produced unique fragmentation spectra that permitted the precise identification of the unknown X-12441 as the 12-HETE isoform (see Text S3 for fragmentation spectra).

#### Scenarios 4–6

Three further scenarios, involving acylcarnitines in connection to mitochondrial β-oxidation enzymes (CARNITINE), bilirubin metabolism (BILIRUBIN), and a connection between ascorbate metabolism and catechol-O-methyltransferase (ASCORBATE), are described in Text S3. Briefly, in the CARNITINE scenario, X-11421 and X-13431 were experimentally confirmed as acylcarnitines containing 10 and 9 carbon atoms, respectively (namely cis-4-decenoyl-carnitine and nonanoyl carnitine). For the BILIRUBIN scenario, the unknown metabolite X-11793 was identified as an oxidized bilirubin variant. Lastly, in the ASCORBATE scenario, X-11593 was predicted and experimentally confirmed as O-methylascorbate. Taken together, we here predicted and experimentally validated the biochemical identity of nine unknown metabolites in six different biochemical scenarios.

## Discussion

We developed and validated a novel integrative approach for the biochemical characterization of “unknown metabolites” from high-throughput metabolomics and genotyping datasets. Our method allows for the functional annotation of previously unidentified metabolites and, as a consequence, enhances the interpretability of metabolomics data in genome-wide association studies and biomarker discovery. For the first time, we systematically evaluated genetic associations of unknown metabolites, thereby discovering seven new loci of metabolic individuality. By classifying a series of unknown metabolites, we gained new insights into the functional interplay between genetic variation and the metabolome both for previously reported and new loci. Furthermore, several of the unknown compounds that we identified as well as their newly associated loci were independently reported in disease-related studies. In the following, we discuss three genetic loci and their associated phenotypes.

### COMT and hepatic detoxification

The first example is a recent biomarker study, where Milburn et al. [34] reported an association of X-11593 with hepatic detoxification. In our GWAS, we find a strong association of X-11593 with the COMT locus, which encodes the *catechol-O-methyltransferase* enzyme. COMT is responsible for the inactivation of catecholamines such as L-dopa and various neuroactive drugs by O-methylation [35]. Following our identification approach, we experimentally confirmed the identity of X-11593 as O-methylascorbate. Notably, O-methylascorbate is a known product of ascorbate (vitamin C) O-methylation by COMT [36], [37]. Thus, our observations establish a link between O-methylascorbate blood levels, common genetic variation in the COMT locus and COMT-mediated liver detoxification processes.

### ACE and hypertension

The second example relates to the ACE gene locus, which is a known risk locus for cardiovascular disease, hypertension and kidney failure. The protein encoded by the ACE locus, *angiotensin-converting enzyme*, is an exopeptidase which cleaves dipeptides from vasoactive oligopeptides, and plays a central role in the blood pressure-controlling renin-angiotensin system [38]. Moreover, the ACE protein is a target for various pharmaceuticals (ACE inhibitors), especially in the treatment of hypertension [39]. In our study, we identified three unknowns as dipeptides (X-14205, X-14208 and X-14478), two of which also associated with the ACE locus. These dipeptides could thus represent novel, interesting biomarkers for the activity of ACE. Moreover, Steffens et al. [11] reported a connection between heart failure and X-11805, which is in close proximity to angiontensin-related peptides in the GGM. This connection might be revisited after a successful identification of X-11805 in a future study.

### UGT1A/ACADM and insulin resistance

The third example is an explorative study to detect biomarkers for insulin sensitivity. Gall et al. [9] reported several known metabolites (most prominently α-hydroxybutyrate) as biomarkers for insulin resistance. They also reported a series of unknown metabolites among their top hits. In the present study, we investigated three of these unknowns: X-11793 associates with UGT1A (*UDP glucuronosyltransferase 1*) and represents a bilirubin-related substance. Moreover, we experimentally validated X-11421 and X-13431, which display a strong association with ACADM (*acyl-Coenzyme A dehydrogenase, C-4 to C-12 straight chain*), as acylcarnitines containing 10 and 9 carbon atoms, respectively. The identification of these latter two unknown metabolites as medium-chain length acylcarnitines is coherent with reports by Adams et al. [40]. The authors found elevated blood plasma acylcarnitine levels in women with type 2 diabetes. Functionally, they attributed this finding to incomplete β-oxidation. Thus, our identification of X-11421 and X-13431 now suggests incomplete β-oxidation as an explanation for the associations found by Gall et al. and implies that acylcarnitines containing 10 and 9 carbon atoms are potential biomarkers for insulin resistance.

### Conclusion

In summary, we integrated high-throughput metabolomics and genotyping data from a large population cohort for elucidating the biochemical identities of unknown metabolites. To this end, we applied metabolomics genome-wide association studies and Gaussian graphical modeling in order to link these unknown metabolites with known metabolic classes and biological processes. For six specific scenarios, we went from systematic hypothesis generation over detailed investigation and identity prediction to direct experimental confirmation. Similar validations may now be undertaken for the remaining predictions that we report in Table S1. Finally, we demonstrated the benefit of our method by discussing several of these newly identified metabolites in the context of existing biomarker discovery studies on liver detoxification, hypertension and insulin resistance.

It is to be noted that our method does not specifically require genotyping data. Even metabolomics measurements alone, analyzed through the GGMs, may provide sufficient information for the classification and even precise identity prediction. The unknowns with GGM evidence but without GWAS hits in Figure 4 as well as the HETE scenario represent examples for this approach.

One limitation of our approach is the requirement for associations with functionally described loci or known metabolites. Certain metabolite groups might thus systematically not be identifiable. For instance, if the identity of a whole class of biochemically related molecules is unknown (which might be due to experimental reasons), then the GGM associations between those compounds will not aid in identity elucidation. The 118 unknown compounds for which we could not derive any classification might represent such cases. Thus, our functionally oriented method should be regarded as a complementary extension to the existing identity determination methods.

Accordingly, our approach can be extended in several directions. It can be combined with method-specific, automated techniques that further exclude sets of metabolites. Previously mentioned methods relying on mass-spectra [15] or chromatographic properties [17] are suitable candidates here. Moreover, the method can be directly transferred to other types of metabolomics datasets not specifically originating from MS experiments, such as NMR-based metabolomics.

Beyond the application to metabolite identification, our study demonstrates the general potential of functional metabolomics in the context of genome-wide association studies. The comprehensive metabolic picture provided by GGMs in combination with GWAS allows for the detailed analysis of metabolic functions, chemical classes, enzyme-metabolite relationships and metabolic pathways.

## Materials and Methods

### Study cohort and data acquisition

We used data from n = 1768 fasting serum samples used in a previously published genome-wide association study on a German population cohort. Details of the sample acquisition and experimental procedures can be found in [5]. Briefly, metabolic profiling was done using ultrahigh-performance liquid-phase chromatography and gas-chromatography separation, coupled with tandem mass spectrometry. The dataset contains a total of 292 known compounds and, in addition to the GWAS study in [5], 225 unknown compounds. Metabolite concentrations were log-transformed since a test of normality showed that in most cases the log-transformed concentrations were closer to a normal distribution than the untransformed values [5]. Genotyping was carried out using the Affymetrix GeneChip array 6.0. For our analyses, we only considered autosomal SNPs passing the following criteria: call rate >95%, Hardy-Weinberg-Equilibrium p-value p(HWE)>10^−6^, minor allele frequency MAF>1%. In total, 655,658 SNPs were left after filtering.

### Genome-wide associations

In order to avoid spurious false positive associations due to small sample sizes, only metabolic traits with at least 300 non-missing values were included and data-points of metabolic traits that lay more than 3 standard deviations off the mean were excluded by setting them to ‘missing’ in the analysis (leaving 273 known and 213 unknown metabolites). Genotypes are represented by 0, 1, and 2 for major allele homozygous, heterozygous, and minor allele homozygous, individuals respectively. We employed a linear model to test for associations between a SNP and a metabolite assuming an additive mode of inheritance. Statistical tests were carried out using the PLINK software (version 1.06) [41] with age and gender as covariates. Based on a conservative Bonferroni correction, associations with p-values<1.6×10^−10^ meet genome-wide significance, corresponding to a significance level of α = 0.05. SNP-to-gene assignments were derived via linkage disequilibrium (LD) from HAPMAP [42]. A SNP was associated with a gene whenever there was at least one other SNP lying in the transcribed region of this gene (that is from 5′UTR to 3′UTR) that displays an r^2^≥0.8 with the query SNP. A detailed description of the GWAS procedure can be found in [5].

Lookups of previously known associations between phenotypes and genetic variants were performed using the GWAS catalog [26]. We list a phenotype with one of our GWAS hits, if the phenotype was reported with at least one SNP that displays an LD r^2^≥0.5 with the respective “lead SNP”. Lookups of eQTLs were performed for all significant SNPs (474, see Dataset S1) using the GTEx database [29]. We applied a p-value cutoff of 2.7×10^−9^, corresponding to a significance level of 0.05 and correction for 474×40,000 tests (the number of SNPs times number of transcripts, conservative estimate). Detailed results up to a p-value of 10^−5^ can be found in Dataset S1.

### Gaussian graphical modeling

For the GGM calculation, we require a full data matrix without missing values. From the original data matrix containing n = 1768 samples and 517 metabolites (thereof 292 knowns and 225 unknowns), we first excluded metabolites with more than 20% missing values (column direction), and then samples with more than 10% missing values (row direction). The filtered data matrix still contained n = 1764 samples with 355 metabolites (217 knowns and 138 unknowns). Remaining missing values were imputed with the ‘mice’ R package [43]. Note that the numbers of metabolites used in the GWAS and in the GGM analysis differ due to specific constraints for the treatment of missing values in the two methods.

Gaussian graphical models are induced by full-order partial correlation coefficients, i.e. pairwise correlations corrected against all remaining (n-2) variables. GGMs are based on linear regressions with multiple predictor variables. When regressing two random variables *X* and *Y* on the remaining variables in the data set, the partial correlation coefficient between *X* and *Y* is given by the Pearson correlation of the residuals from both regressions. Since our dataset contains more samples than variables, full-order partial correlations can be conveniently calculated by a matrix inversion operation. A significance cutoff of α = 0.05 with Bonferroni correction was applied. A detailed description of the GGM calculation procedure can be found in [21].

Age, gender and SNP effects were removed by adding the respective variables and SNPs states to the data matrix. For each pair of variables under investigation, Gaussian graphical models remove the effects of all remaining variables on this correlation (due to the above-mentioned linear regression approach). That is, adding a variable to the data matrix will automatically result in the removal of confounding effects of this variable on the correlations of all other variables. Note that age, gender and SNPs were not investigated as an actual node in the network but merely used for the correction procedure. For the later analysis steps, we then only considered metabolite-metabolite edges in the network. SNP states were coded as numerical values of 0, 1 and 2 (see previous section), such that the linear regressions that underlie the GGM correspond to an additive genetic model (cf. [5]). Gender represents a “dummy variable” [44] in the linear regression model which only takes values of 1 (male) and 0 (female).

### Metabolic pathway model and functional annotations

Metabolic reactions were imported from three independent human metabolic reconstruction projects: (1) H. sapiens Recon 1 from the BiGG databases [45], (2) the Edinburgh Human Metabolic Network (EHMN) reconstruction [46] and (3) the KEGG PATHWAY database [47] as of January 2012. We attempted to create a highly accurate mapping between the different metabolite identifiers of the respective databases, in order to ensure the identity of each compound in our list. Entries referring to whole groups of metabolites, such as “phospholipid”, “fatty acid residue” or “proton acceptor” were excluded from our study. Furthermore, we did not consider metabolic cofactors such as “ATP”, “CO_2_”, and “SO_4_” etc. in our analysis, since such metabolites unspecifically participate in a plethora of metabolic reactions. For each enzyme catalyzing one or more reactions in our pathway model, we retrieved functional annotations from two independent sources: (i) GO-Terms from the Gene Ontology [48] and (ii) enzyme pathway annotations from the KEGG PATHWAY database [47].

All imported metabolic pathways along with metabolite database identifiers, excluded compounds and pathway annotations can be found in Dataset S1.

## Supporting Information

Dataset S1 ZIP archive containing Excel sheets and .graphml files for the GGM and GWAS results, as well as detailed pathway annotations.
(ZIP)

Figure S1 GGM sub-network with bilirubin variants.
(PDF)

Table S1 Systematic classifications for 106 unknown metabolites.
(XLS)

Text S1 Detailed GGM modularity analysis results.
(PDF)

Text S2 Assessment of the majority voting classification approach.
(PDF)

Text S3 Supporting experimental data and description of the unknown identification scenarios CARNITINE, BILIRUBIN, and ASCORBATE.
(PDF)
