# Supplementary material for: Mining the Unknown: A Systems Approach to Metabolite Identification Combining Genetic and Metabolic Information
Source: PLoS Genet. 2012 Oct 18;8(10):e1003005. doi: 10.1371/journal.pgen.1003005 (PMC3475673; doi:10.1371/journal.pgen.1003005)

FIGURE S1 – BILIRUBIN-CENTERED GGM SUBNETWORK

The following figure contains a high-scoring GGM subnetwork around three bilirubin stereoisoforms containing 7 unknown metabolites. For a detailed description of this network, please see main manuscript.

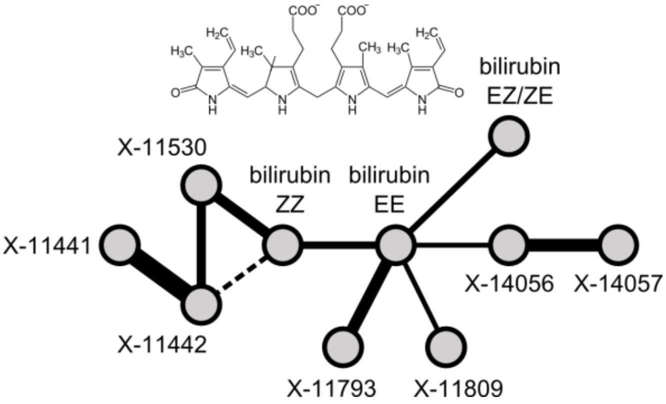

Supplement: Figure S1 — GGM sub-network with bilirubin variants. (PDF) [file pgen.1003005.s002.pdf]
