# Supplementary material for: Mining the Unknown: A Systems Approach to Metabolite Identification Combining Genetic and Metabolic Information
Source: PLoS Genet. 2012 Oct 18;8(10):e1003005. doi: 10.1371/journal.pgen.1003005 (PMC3475673; doi:10.1371/journal.pgen.1003005)
Supplement: Text S1 — Detailed GGM modularity analysis results. (PDF) [file pgen.1003005.s004.pdf]

TEXT S1 – GGM MODULARITY

In the following, we investigated whether our GGM inferred from metabolomics data displays a modular structure with respect to the main metabolic classes in the dataset. Intuitively, the modularity measure Q compares the within-class edges with the edges to the rest of the network. The more edges there are within each class in comparison to the other classes, the higher Q will be. In order to assess the statistical significance of the result, we additionally calculated Q for 10^5 randomized GGM networks (random edge rewiring). For a detailed description of the modularity calculation method and further results on a different metabolite panel, we refer the reader to Krumsiek et al. [1].

We excluded the unknowns in this analysis, as it obviously makes no sense to treat them as a single class in this integrated analysis. For the original GGM, we obtain a modularity of $Q = 0.389$, whereas the random networks yield $Q = -0.0041 \pm 0.0222$ (corresponding to a z-score of $z = 17.71$). This result indicates a strong modular organization of the GGM, where metabolites are mainly connected to other metabolites from the same class, and only few edges connect metabolites of different classes.

Class-wise modularity

The following figure depicts the fraction of edges going from each class (row) to each other class (column). For example, the cell in the second row and third column encodes the fraction edges going from carbohydrates to amino acids in relation to the total number of carbohydrate-associated edges. Note that since the classes are not equally large, this matrix is not symmetric. Adding the unknowns as an additional class to this analysis further emphasized their high abundance in all metabolic classes of our measured panel.

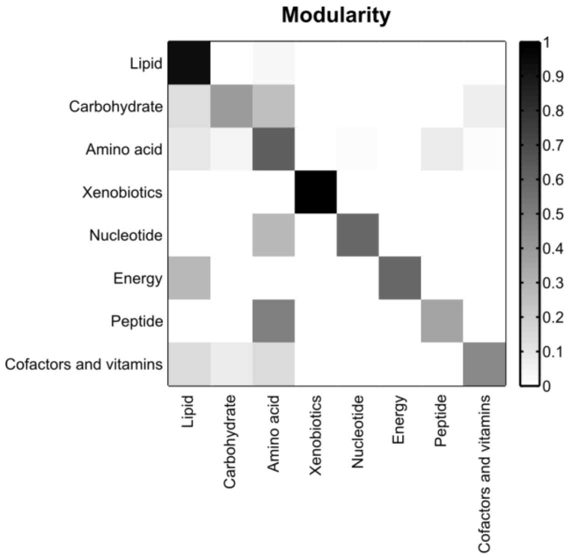

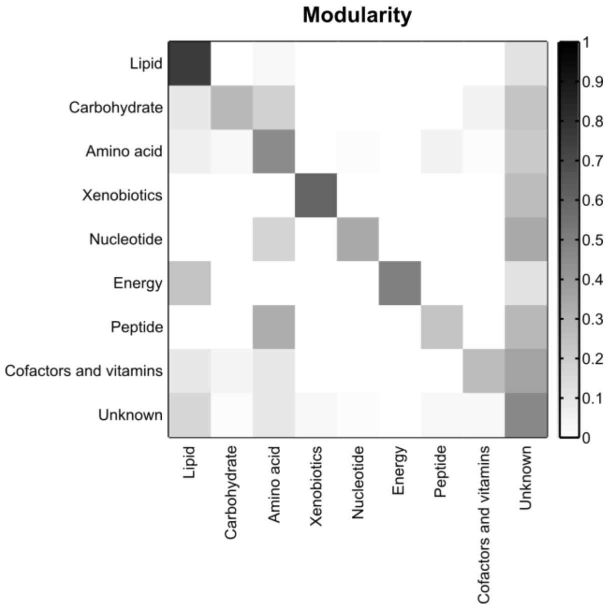

References

1. Krumsiek J, Suhre K, Illig T, Adamski J, Theis FJ (2011) Gaussian graphical modeling reconstructs pathway reactions from high-throughput metabolomics data. BMC Syst Biol 5: 21.
