# Supplementary material for: Mining the Unknown: A Systems Approach to Metabolite Identification Combining Genetic and Metabolic Information
Source: PLoS Genet. 2012 Oct 18;8(10):e1003005. doi: 10.1371/journal.pgen.1003005 (PMC3475673; doi:10.1371/journal.pgen.1003005)
Supplement: Text S2 — Assessment of the majority voting classification approach. (PDF) [file pgen.1003005.s005.pdf]

TEXT S2 – MAJORITY VOTING USING GGM NEIGHBORHOODS

We determined whether the local neighborhood of a metabolite in the GGM can be used to correctly predict its metabolic class by proceeding as follows: Each known metabolite is annotated with one out of eight *super-pathway* annotations, *Carbohydrate*, *Lipid*, *Nucleotide*, *Amino acid*, *Xenobiotics*, *Energy*, *Peptide* and *Cofactors and vitamins* (see Methods). A majority voting approach was implemented, where each (known) metabolite is assigned to the pathway that occurs most frequently amongst its direct GGM neighbors (neighborhood size=1), and then determining whether this indeed corresponds to the true pathway of the metabolite. In case of a tie, i.e. an identical number of neighbors from two or more different pathways, we judge in favor of the classifier. This means, if the correct class is present among the tie classes, we will count this as a true positive. For the unknown classification later on, unknowns that did not show any link to a known metabolite (neither as direct neighbors nor for more than one network step) were excluded from the analysis. We cannot make any prediction, true or false, for these metabolites.

For the known metabolites, this approach yields a classifier quality of F_1=0.718. The F_1-measure represents a quantitative tradeoff between sensitivity and specificity (see methods below). In order to objectively evaluate classification performance, we generated 10^8 randomly rewired GGM networks and recalculated the majority predictions. No random sample achieved an F_1 score equal to or greater than the real GGM, revealing classification abilities far beyond random ($p<10^{-8}$, see Figure 1 below). It is to be noted at this point, that the actual quality of our classifier might be even higher, since GGM connections between different classes should not always be considered false positive. As an example, metabolites assigned to different inherently related classes such as “amino acid” and “peptide” might actually belong to the same pathway. A classical hypergeometric enrichment analysis [1] amongst the neighbor classes of a node in the network is not appropriate, since the inherent sparseness of a GGM is not compatible with the null model behind an enrichment approach. While obviously majority voting is amongst the simplest possible classifiers, it is easy to implement and performs well for the task at hand.

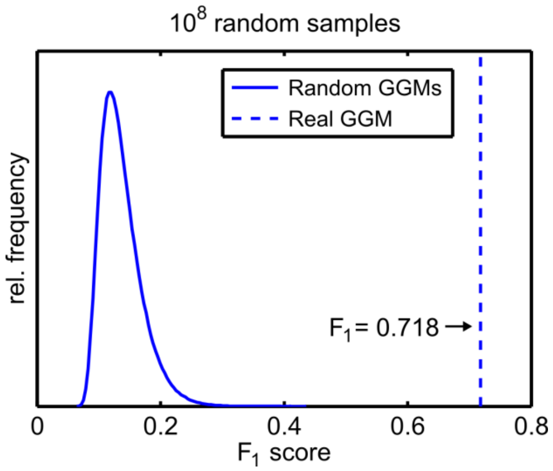

Figure 1: F_1 score for the GGM compared to 10^8 rewired networks.

Detailed results

The following table shows detailed classification results for each class. A definition of sensitivity, specificity and the F1 score is given in the "Statistical methods." section below. In general, we observe good classification properties for most classes. Poor classification of carbohydrates is probably due to their involvement in many distinct metabolic pathways. For the peptide class, the low sensitivity is due to many links of peptide metabolites with amino acids (which is not necessarily a false result but will be penalized in our simplified classifier).

	Sensitivity	Specificity	F_1
**Lipid**	0.944	0.903	0.923
**Carbohydrate**	0.429	0.375	0.400
**Amino acid**	0.795	0.705	0.747
**Xenobiotics**	1.000	1.000	1.000
**Nucleotide**	0.750	1.000	0.857
**Energy**	0.600	1.000	0.750
**Peptide**	0.250	1.000	0.400
**Cofactors and vitamins**	0.625	0.714	0.667
Ø	0.674	0.837	
**Macro-averaged F_1**			0.718

Choice of the null model.

Note that edge rewiring is not the only possible null model to test a network property for statistical significance. Another prominent approach is node shuffling, where instead of changing the network topology we randomly reassign the node labels. A comparison of the discrimination quality using node shuffling corresponds to the question "Are the node classes arranged in a specific pattern on the network, or is this signal just by chance?" On the other hand, edge rewiring addresses the question "Does the network structure contain particular information on similar nodes, or are they just randomly wired?" We here specifically wanted to address the second question.

Statistical methods.

In order to evaluate whether the class of a metabolite can be properly predicted by the classes of its direct GGM neighbors, we calculated sensitivity, specificity and the F_1 score for each metabolic class (i.e. each of the eight *super-pathway* annotations). Therefore, we computed a confusion matrix $C \in \mathbb{N}_+^{8\times 8}$ where each element c_{ij} counts the number of neighbors of class j for each metabolite of class i. In other words, we count how many times the actual (correct) class i was predicted to be class j by a GGM neighbor. For a given class i we then define true positives $\mathrm{TP}_i := C_{ii}$, false positives $\mathrm{FP}_i := \sum_{j\neq i} C_{ji}$, false negatives $\mathrm{FN}_i := \sum_{j\neq i} C_{ij}$, and true negatives $\mathrm{TN}_i := \sum_{j\neq i,k\neq i} C_{jk}$.

The class-wise sensitivity s_i and specificity p_i is given by

$$s_i := \frac{\mathrm{TP}_i}{\mathrm{TP}_i + \mathrm{FN}_i}, \qquad p_i := \frac{\mathrm{TN}_i}{\mathrm{TN}_i + \mathrm{FP}_i}$$

Finally, the macro-averaged F_1 score is defined as the harmonic mean of the average sensitivity $\bar{s} = \sum_{i=1}^{8} s_i \,/\, 8$ and average specificity $\bar{p} = \sum_{i=1}^{8} p_i \,/\, 8$ [2]:

$$F_1 = 2 \cdot \frac{\bar{s} \cdot \bar{p}}{\bar{s} + \bar{p}}$$

To assess the significance of our majority voting class predictor, we performed graph randomization by edge rewiring of the GGM. During the rewiring process we randomly pick two edges from the network and exchange the target nodes of each edge. If a rewiring step results in already-existing edges, the step is repeated with two new randomly chosen edges. In order to achieve sufficient randomization, this operation is repeated $5 \cdot e$ times, where e represents the number of edges in the graph [3].

Possible bias of the edge rewiring procedure.

We performed an additional analysis to exclude a systematic underestimation of the classification properties during edge rewiring. To this end, we performed the same procedure of F_1 calculation for one real vs. a number of randomized networks, but with initially reshuffled node labels (thereby destroying the original ordering of nodes). Figure 2 below shows the empirical p-values of these node-shuffled networks under the null model of edge rewiring. As can be seen from the histogram, empirical p-values around 0.25 to 0.45 demonstrate that the randomized F_1 scores distribute around the original randomized F_1 scores of the analyses above. There is no systematic under- or over-estimation of classification quality per se. In other words, in a network without a signal, the edge rewiring approach also does not claim to find a signal; which would be a problematic method bias.

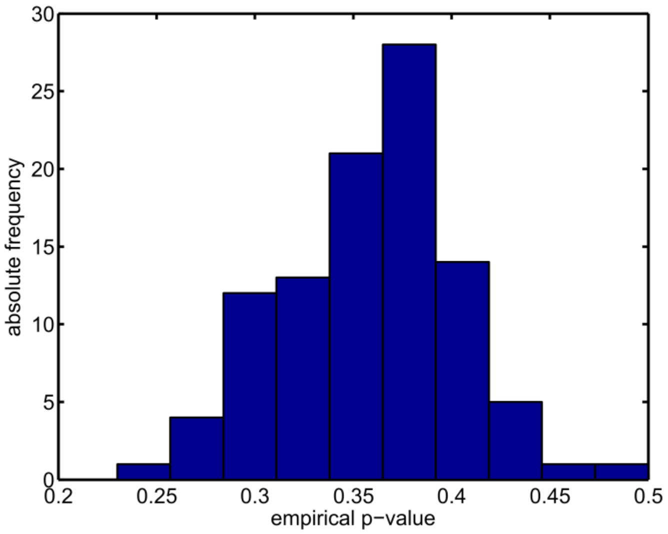

Figure 2: Empirical p-values determined by edge-rewiring on 100 networks with reshuffled node labels. Each p-value is based on 100 rewired networks. Values from ~0.25 to 0.45 indicate that there is no systematic over- or underestimation of F_1 scores due to edge rewiring.

References

1. Subramanian, A., et al., *Gene set enrichment analysis: a knowledge-based approach for interpreting genome-wide expression profiles.* Proc Natl Acad Sci U S A, 2005. **102**(43): p. 15545-15550.
2. Van Rijsbergen, C.J., *Information Retrieval, 2nd edition*. 1979: Dept. of Computer Science, University of Glasgow.
3. Wong, P., et al., *An evolutionary and structural characterization of mammalian protein complex organization.* BMC Genomics, 2008. **9**: p. 629.
