# Supplementary material for: Mining the Unknown: A Systems Approach to Metabolite Identification Combining Genetic and Metabolic Information
Source: PLoS Genet. 2012 Oct 18;8(10):e1003005. doi: 10.1371/journal.pgen.1003005 (PMC3475673; doi:10.1371/journal.pgen.1003005)
Supplement: Text S3 — Supporting experimental data and description of the unknown identification scenarios CARNITINE, BILIRUBIN, and ASCORBATE. (PDF) [file pgen.1003005.s006.pdf]

TEXT S3 – FURTHER EXPERIMENTAL VALIDATIONS AND DATA

Fragmentation spectra for 4-androsten-3β,17β-disulfate, α-glutamyltyrosine (α-Glu-Tyr), phenylalanylphenylalanine (Phe-Phe), and 12-hydroxyeicosatetraenoic acid (12-HETE)

We here provide the fragmentation spectra for the androstene disulfate, the two dipeptide examples and 12-HETE from the main manuscript. The mass to charge ratio given for the unknown represents the parent mass peak. This ion was also used for quantification.

X-11244 (224.2 m/z), RI = 3729, neg. mode

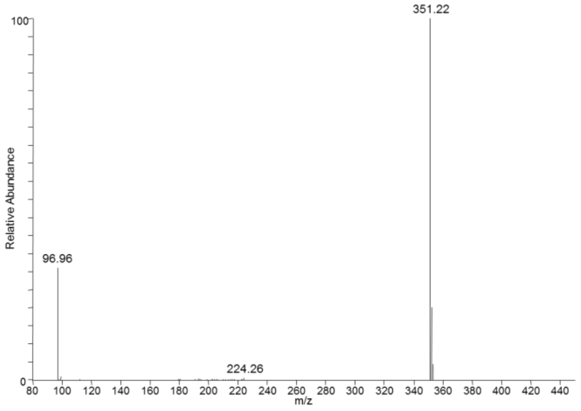

Standard 4-androsten-3β,17β-disulfate, RI = 3735

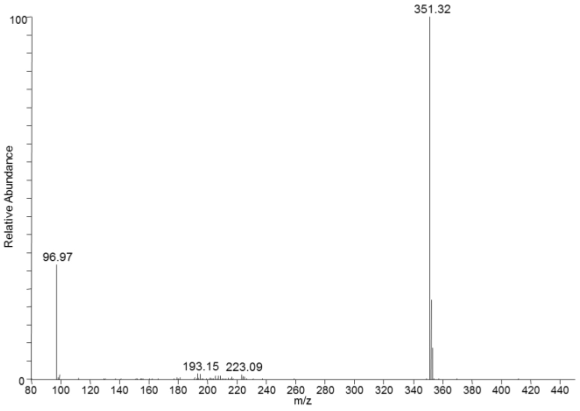

X-14205 (311.1 m/z), RI = 1820, pos. mode

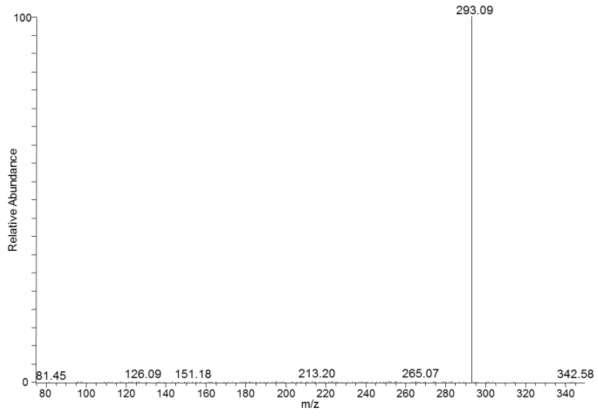

Standard α-Glu-Tyr, RI = 1810

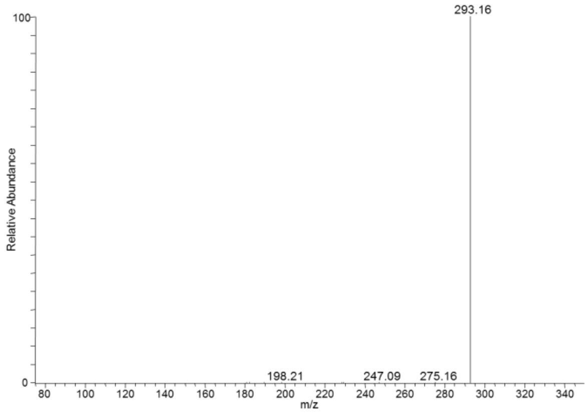

X-14478 (313.2 m/z), RI = 3377, pos. mode

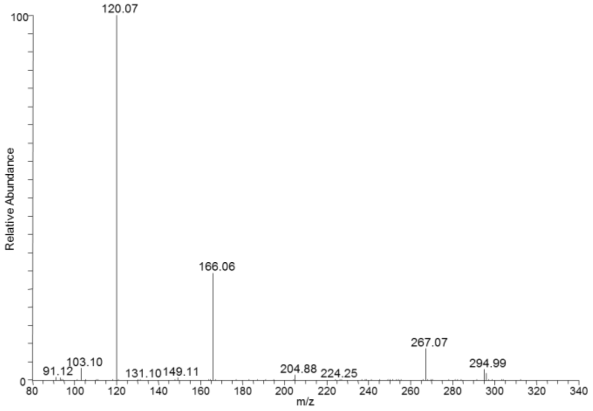

Standard Phe-Phe, RI = 3317

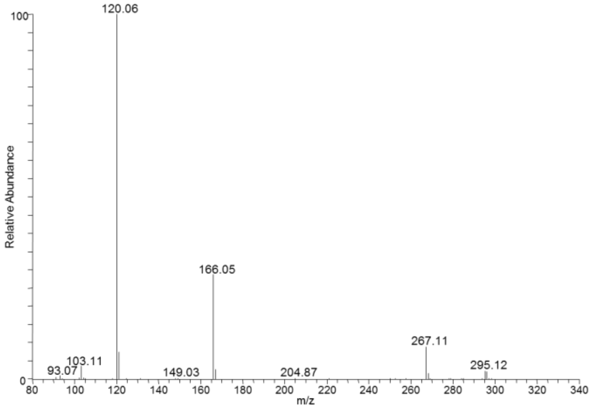

X-12441 (319.4 m/z), RI = 5301, neg. mode

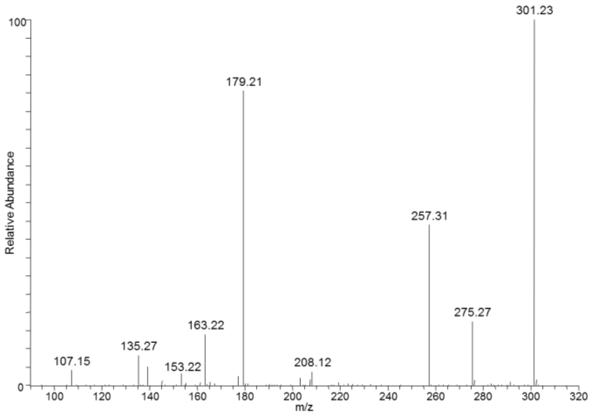

Standard 12-HETE, RI = 5295

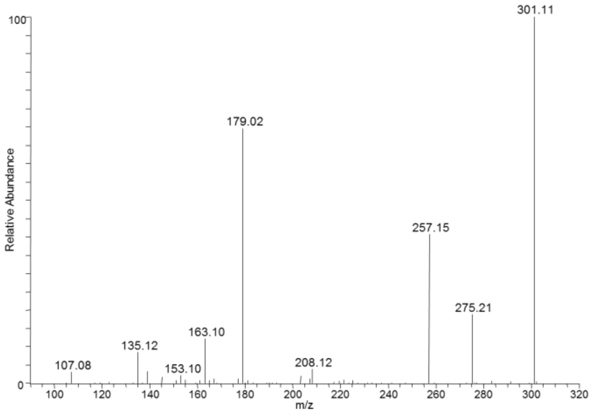

Scenario CARNITINE – X-11421 and X-13431 represent medium-chain acylcarnitines

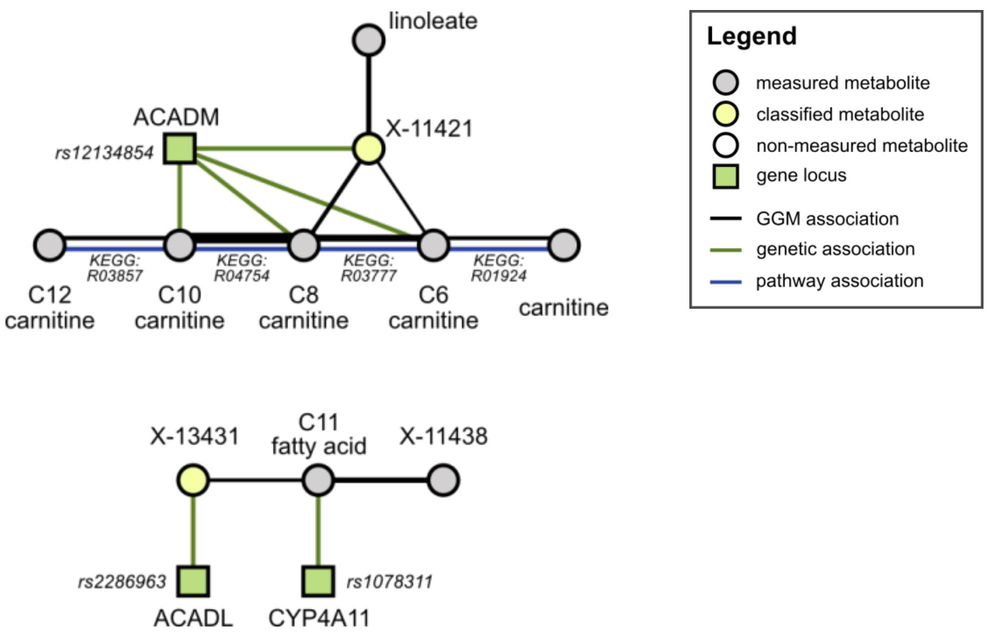

In the CARNITINE scenario, we investigated two specific unknowns that, on the one hand, display associations with fatty acid derivatives, in particular with acylcarnitines, and, on the other hand, associate with enzymes of the *acyl-coenzyme A dehydrogenase*, ACAD, class. Acylcarnitines represent a transport form of fatty acids tagged for mitochondrial transport and subsequent β-oxidation [1]. In previous studies we already demonstrated (a) strong GGM edges between carnitine species with a carbon atom difference of two [2], and (b) genetic associations between various acylcarnitines and loci encoding for β-oxidation related ACAD enzymes.

The first unknown metabolite, X-11421, shares significant GGM edges with C8 and C6 carnitines and further associates with ACADM, the ACAD enzyme for medium-chain length fatty acyl residues. In the context of our previous findings and considering the mass peak of X-11421 (314.2 m/z, positive mode), we therefore hypothesized that X-11421 is a medium-chain length carnitine with 10 carbon atoms.

Matching our computational prediction, this unknown has indeed been experimentally identified as *cis-4-decenoyl-carnitine*, a carnitine with 10 carbon atoms and an ω-6 double bond, by testing the pure compound. It has to be noted that carnitines shift elution times dramatically in relation to their RI markers on the analytical platform used in this work. The cis-4-form was confirmed in a spiking experiment in a well characterized human plasma sample, which was run with original samples.

X-11421 (314.2 m/z), RI = 4575, pos. mode

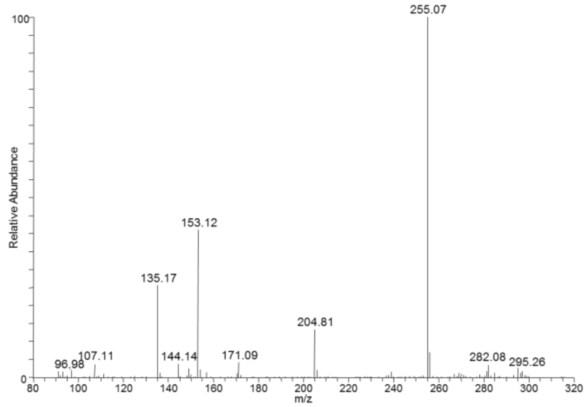

Standard cis-4-decenoyl carnitine, RI = 4404

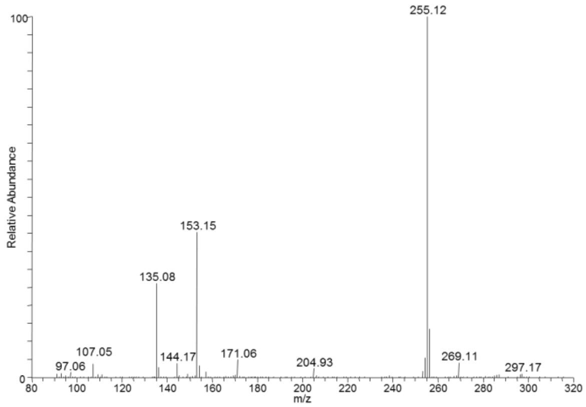

The second unknown metabolite, X-13431, is linked to a C11 free fatty acid in the GGM, and with the ACADL locus. In a previous study, this locus has been shown to associate with C9 carnitine levels [3]. This observation together with the molecule mass peak detected for the unknown (302.3 m/z, positive mode) make C9 carnitine a good candidate for X-13431.

Our prediction is experimentally confirmed by the fragmentation of X-13431 as the molecule produces fragments shared by mid- and long-chain acylcarnitines and several neutral losses (loss of 59 m/z and 161 m/z) that are highly diagnostic of carnitines. With respect to chromatography, X-13431 elutes between C8 and C10 carnitines, thus further supporting the hypothesized C9 carnitine. The accurate mass of 301.22476±0.0015 Da determined for X-13431 corresponds to the molecular formula $C_{16}H_{31}NO_4$ and, thus, also matches C9 carnitine. Due to the lack of a commercial source for pure C9 carnitine, the final confirmation of the predicted chemical identity by testing the pure compound is still pending.

X-13431 (302.3 m/z), RI = 4230, pos. mode

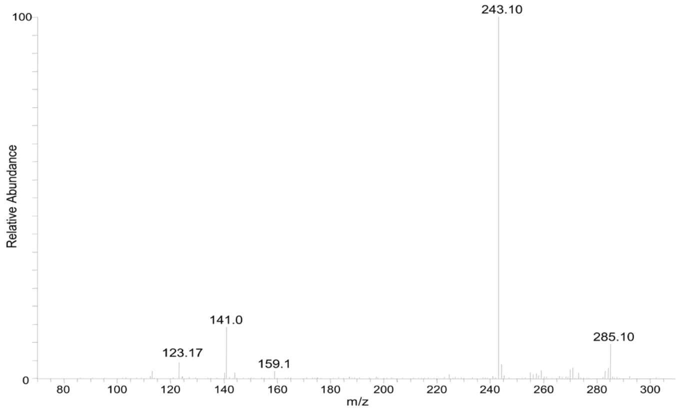

Scenario BILIRUBIN – X-11793 represents an oxidized bilirubin variant

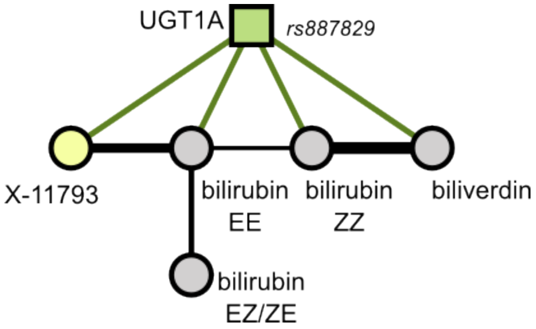

In the BILIRUBIN scenario, we focused on the unknown metabolite X-11793 (601.1 m/z, positive mode), which shares a GGM edge with a specific bilirubin stereoisomer (EE) and associates with the UGT1A locus encoding for the enzyme *UDP glucuronosyltransferase 1 family, polypeptide A*. The bilirubin stereoisomers and biliverdin, which shows close proximity in the GGM, are degradation products of heme, the oxygen-carrying prosthetic group contained in hemoglobin [4]. For further metabolization and excretion, the very insoluble bilirubin must be transformed into soluble derivatives. Glucuronidation of bilirubin represents the main mechanism for this transformation in the human metabolism. The transformation is mainly catalyzed by an enzyme encoded at the UGT1A1 locus [5,6], matching the observations in our data that X-11793 and three of the four degradation products display genetic associations with the UGT1A locus.

Since X-11793 is embedded in the biochemical and genetic network of bilirubin derivatives and also shares their association with the UGT1A locus, we assumed that X-11793 represents a further bilirubin derivative. Moreover, the mass difference between bilirubin and X-11793 is 15.9, which might correspond to the addition of oxygen. We therefore predicted X-11793 to be an (ep)oxidized bilirubin as a possible result of bilirubin oxidation mediated by cytochrome P450. Such oxidation processes have been suggested as alternative routes for the metabolization of bilirubin besides glucuronidation previously [7,8,9].

From an experimental perspective, the neutral accurate mass of X-11793 of 600.25859 Da corresponding to $C_{33}H_{36}N_4O_7$, perfectly matches the formula for the predicted (ep)oxidized bilirubin variant. The fragmentation pattern produced by the unknown molecule further supports the hypothesis: Bilirubin generates fragments with 285 m/z and 299 m/z corresponding to a cleavage of the central C-C bond of the molecule. If the hypothesized ep(oxidized) bilirubin broke at the same position, it would produce fragments with 301 m/z and 299 m/z accordingly, which both occur in the fragmentation spectrum of X-11793. The final confirmation of the prediction by running pure epoxidized bilirubin is still pending due to the lack of commercial sources of the pure substances.

X-11793 (601.1 m/z), RI = 3634, pos. mode

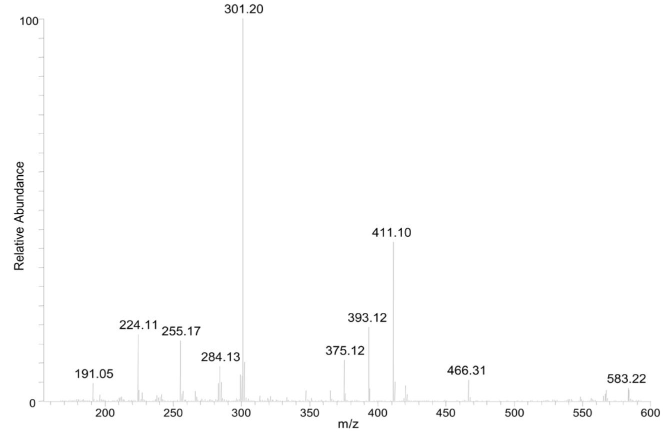

epoxidized bilirubin

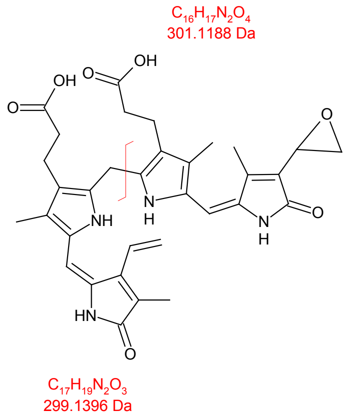

X-11793 identified as (ep)oxidized bilirubin might represent an interesting additional biomarker for the efficacy of heme degradation processes, which plays an important role in various diseases. Serum concentrations of bilirubin as well as the UGT1A locus encoding the enzyme mainly responsible for the degradation of bilirubin are not only associated with bilirubin turnover-related syndromes such as jaundice but also with different cancer variants and coronary heart disease (CHD) [5,10,11,12,13]. While jaundice is caused by high bilirubin concentrations, bilirubin has proven to be an effective antioxidant [14], which might explain the association found between reduced risk of CHD and various forms of cancer with higher bilirubin concentrations.

Scenario ASCORBATE – X-11593 represents O-methylascorbate

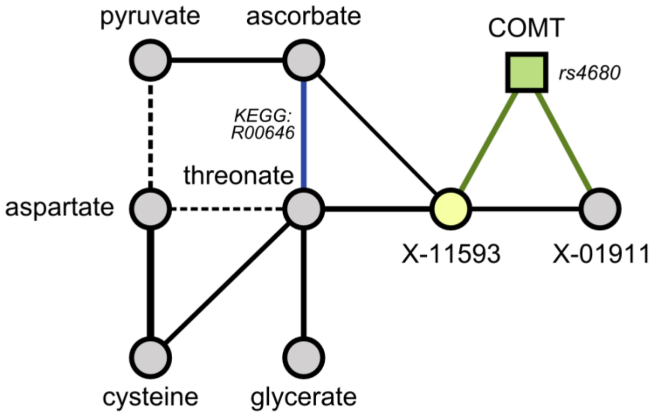

In the ASCORBATE scenario, we investigated the unknown X-11593 (189.2 m/z, negative mode), which is close to threonate, ascorbate and related substances in the GGM. These metabolites are tightly interconnected in the ascorbate (vitamin C) pathway. Furthermore, we found significant associations of X-11593 with SNPs in the gene encoding *catechol-O-methyltransferase* (COMT), an enzyme relevant for

the inactivation and degradation of many drugs. COMT O-methylates molecules with catechol-like structures.

Since, according to the GWAS, X-11593 is probably a substrate or a product of O-methylation, we determined the mass differences to the known metabolites neighboring X-11593, namely ascorbate and threonate. While the mass difference of X-11593 and threonate is 54, X-11593 and ascorbate show a mass difference of 14, which corresponds to the addition of a methyl moiety. Moreover, in ascorbate, the double bond within the 5-ring with its two hydroxyl moieties could "mimic" the corresponding planar substructure in catechol, on which COMT is usually working. Finally, the methylation of ascorbate through the catalysis of COMT has already been shown experimentally [15]. These observations make O-methylated ascorbate derivatives (most probably 2-O-methylascorbate) good candidates for X-11593.

From an experimental perspective, our hypothesis is supported by the accurate neutral mass of 190.04787 Da determined for X-11593. Based on the accurate mass, the molecular formula for X-11593 is $C_7H_{10}O_6$ matching our prediction. The retention time of X-11593 shows a slight shift compared to the time for ascorbate. This shift matches the shift expected for adding a methyl group. Moreover, X-11593's primary fragment loss is 60 m/z, which is the same as for ascorbate. The loss of 15 m/z, also seen for X-11593, is typical for phenols substituted with a –OH and -OCH_3. Due to the lack of a commercial source for 2-O-methylascorbate the confirmation through the spectrum of the pure substance is still pending.

X-11593 (189.2 m/z), RI = 790, neg. mode

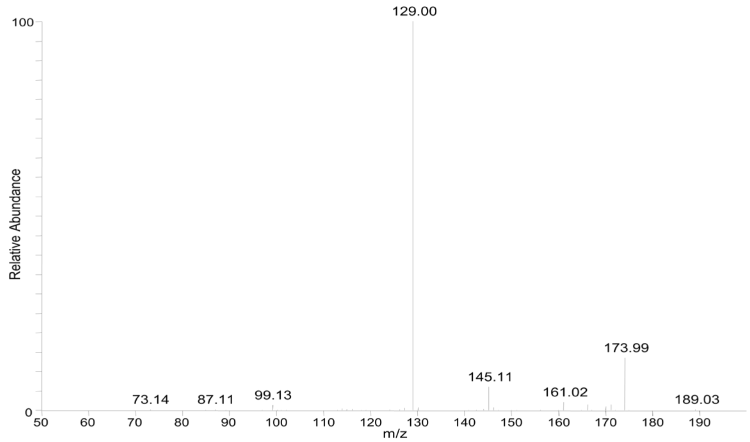

BIBLIOGRAPHY

1. EATON, S. Control of mitochondrial beta-oxidation flux. **Prog Lipid Res**, v. 41, n. 3, p. 197-239, 2002.

2. KRUMSIEK, J. et al. Gaussian graphical modeling reconstructs pathway reactions from high-throughput metabolomics data. **BMC Syst Biol**, v. 5, p. 21, 2011.

3. ILLIG, T. et al. A genome-wide perspective of genetic variation in human metabolism. **Nat Genet**, v. 42, n. 2, p. 137-141, 2010.

4. BODEN, G. et al. Metabolism. In: ______ **Textbook of Hepatology**. [S.l.]: Blackwell Publishing Ltd, 2008. p. 129-249.

5. TUKEY, R. H.; STRASSBURG, C. P. Human UDP-glucuronosyltransferases: metabolism, expression, and disease. **Annu Rev Pharmacol Toxicol**, v. 40, p. 581-616, 2000.

6. BOSMA, P. J. Inherited disorders of bilirubin metabolism. **J Hepatol**, v. 38, n. 1, p. 107-117, 2003.

7. ABU-BAKAR, A.; MOORE, M. R.; LANG, M. A. Evidence for induced microsomal bilirubin degradation by cytochrome P450 2A5. **Biochem Pharmacol**, v. 70, n. 10, p. 1527-1535, 2005.

8. ZACCARO, C. et al. Role of cytochrome P450 1A2 in bilirubin degradation Studies in Cyp1a2 (-/-) mutant mice. **Biochem Pharmacol**, v. 61, n. 7, p. 843-849, 2001.

9. KUMAR, D.; DE VISSER, S. P.; SHAIK, S. Multistate reactivity in styrene epoxidation by compound I of cytochrome p450: mechanisms of products and side products formation. **Chemistry**, v. 11, n. 9, p. 2825-2835, 2005.

10. KIBRIYA, M. G. et al. A pilot genome-wide association study of early-onset breast cancer. **Breast Cancer Res Treat**, v. 114, n. 3, p. 463-477, 2009.

11. ROTHMAN, N. et al. A multi-stage genome-wide association study of bladder cancer identifies multiple susceptibility loci. **Nat Genet**, v. 42, n. 11, p. 978-984, 2010.

12. DJOUSSÉ, L. et al. Total serum bilirubin and risk of cardiovascular disease in the Framingham offspring study. **Am J Cardiol**, v. 87, n. 10, p. 1196--200; A4, 7, 2001.

13. BOSMA, P. J. et al. UGT1A1*28 allele and coronary heart disease: the Rotterdam Study. **Clin Chem**, v. 49, n. 7, p. 1180-1181, 2003.

14. BARANANO, D. E. et al. Biliverdin reductase: a major physiologic cytoprotectant. **Proc Natl Acad Sci U S A**, v. 99, n. 25, p. 16093-16098, 2002.

15. BOWERS-KOMRO, D. et al. Confirmation of 2-O-methyl ascorbic acid as the product from the

enzymatic methylation of L-ascorbic acid by catechol-O-methyltransferase. **Int J Vitam Nutr Res.**, 52, n. 2, 1982.

16. SUHRE, K. et al. Human metabolic individuality in biomedical and pharmaceutical research. **Nature**, v. 477, n. 7362, p. 54-60, 2011.
